# Land Use Shapes the Rhizosphere Microbiome and Metabolome of Naturally Growing *Barbarea vulgaris*

**DOI:** 10.3390/metabo15110684

**Published:** 2025-10-22

**Authors:** Emoke Dalma Kovacs, Melinda Haydee Kovacs

**Affiliations:** Research Institute for Analytical Instrumentation, National Institute for Research and Development in Optoelectronics INOE 2000, Donath 67, 400293 Cluj-Napoca, Romania; dalmaemokekovacs@gmail.com

**Keywords:** habitat type, PLFA profiling, mass spectrometry, plant–microbe interactions, carbon-nitrogen cycling, pathway

## Abstract

**Background:** Land use change fundamentally alters soil microbial communities and biochemical processes, yet the integrated effects on rhizosphere microbiome–metabolome networks remained poorly understood. **Objective:** This study investigated land uses as forest, grassland and intermediary edge shape the rhizosphere biochemical networks of naturally grown *Barbarea vulgaris*. **Methods:** Rhizosphere soils of *Barbarea vulgaris* were analysed for microbial community structure abundance, and metabolomic profile applying phospholipid fatty acid (PLFA) profiling and mass spectrometric untargeted metabolomics (GC–MS/MS and MALDI–TOF/TOF MS). These were coupled with co–inertia analysis to assess microbiome–metabolome interactions. **Results:** Microbial community analysis revealed significant effects of land use on bacterial community structure (G+/G−, *p* < 0.001). Untargeted metabolomics identified 248 metabolites, of which 161 were mapped to KEGG pathways. Amino acids and derivatives (21.1%) followed by organic acids (16.8%) were the most representative among identified metabolites. Pathway enrichment analysis revealed coordinated reprogramming of central carbon and nitrogen metabolism across land use gradients, particularly in the amino acid metabolism, TCA cycle, and glycolysis/gluconeogenesis pathways. Microbiome–metabolome coupling analysis revealed distinct correlation patterns between microbial phenotypes and metabolite classes, with forest environments showing the strongest biochemical network integration (RV = 0.91). Edge habitats presented intermediate signatures, supporting their role as transitional zones with unique biochemical properties. **Conclusions:** The environmental context fundamentally shapes rhizosphere biochemical network organization through coordinated shifts in bacterial community structure and metabolic pathway activity. These habitat-specific metabolic signatures suggest that land use change triggers adaptive biochemical responses that may influence plant performance and ecosystem functioning across environmental gradients.

## 1. Introduction

The rhizosphere represents a dynamic interface where plant roots, soil microorganisms, and chemical compounds interact in complex networks that fundamentally influence plant health, nutrient acquisition, and ecosystem functioning [1]. This narrow zone of soil surrounding plant roots harbors diverse microbial communities whose composition and metabolic activities are shaped by root exudates, creating unique biochemical environments distinct from those of bulk soil [2]. The rhizosphere microbiota facilitates critical processes, including nutrient solubilization, pathogen suppression, stress tolerance, and plant growth promotion, through the production of specialized metabolites [3]. Understanding these intricate plant–microbiota–metabolite interactions has become increasingly important for elucidating the mechanisms underlying plant adaptation, ecosystem resilience, and sustainable management practices in an era of environmental change and intensifying land use pressures.

Land use patterns, particularly the distinction between forest and grassland ecosystems, profoundly influence plant rhizosphere microbial communities and metabolite profiles through multiple interconnected pathways [4]. Wang et al. [5] reported that forest environments typically maintain stable soil conditions with relatively high organic matter contents and diverse litter inputs. These conditions support complex microbial assemblages dominated by fungi and specialized bacterial taxa. In contrast, Liu et al. [6] reported that grassland ecosystems are characterized by more dynamic soil conditions with frequent disturbance, lower organic matter accumulation, and microbial communities adapted to rapid nutrient cycling and root turnover. These contrasting land uses create divergent selective pressures that shape root exudate composition, soil chemistry, and nutrient cycling patterns, ultimately determining the structure and function of rhizosphere microbiomes [7]. Furthermore, Sokol et al. [8] and Gupta et al. [9] reported that differences in vegetation diversity, soil pH, moisture regimes, and carbon input quality between forests and grasslands establish unique metabolic niches that influence microbial metabolite production and plant–microbe signalling pathways. Such land-use-driven variations in rhizosphere communities have cascading effects on plant performance, stress responses, and ecological interactions according to Yusuf et al. [10] and Chauhan et al. [11], highlighting the critical importance of understanding these relationships for predicting ecosystem responses to land use change.

Despite the increasing recognition of the importance of rhizosphere microbiota and metabolites in ecology [12], significant knowledge gaps persist regarding how land use patterns influence these critical plant–soil interactions [3], particularly in naturally occurring plant populations. Most existing research has focused on agricultural systems or controlled greenhouse conditions [1,13], limiting our understanding of how wild plant responses to natural environmental gradients. *Barbarea vulgaris* (Brassicaceae) serves as an ideal model to address this gap because it naturally occurs across multiple land use types within the same region, enabling direct habitat comparisons while controlling for regional factor. Additionally, its widespread distribution across Europe facilitates broader ecological inference. Specifically, there remains limited knowledge about how the same plant species growing in adjacent but contrasting land uses develops distinct rhizosphere communities and metabolite profiles and whether these differences represent adaptive responses to local environmental conditions [14] or merely passive responses to altered soil properties, as concluded by Luo et al. [15]. Furthermore, the temporal stability of land use effects on rhizosphere communities remains poorly understood, as does the relative importance of direct soil effects versus indirect effects mediated through changes in plant physiology and root exudation patterns [1]. The mechanistic links between specific microbial taxa [3], their metabolic products [12], and plant performance outcomes in different land use contexts require further elucidation to understand the functional significance of the observed community differences. Additionally, most studies have examined either microbial communities or metabolite profiles in isolation, with few investigations integrating both approaches to provide comprehensive insights into rhizosphere function [16,17]. The role of plant-by-environment interactions in shaping rhizosphere responses to land use remains largely unexplored, despite potential implications for plant adaptation and population differentiation, as observed by Resinula et al. [18] and Luo et al. [19]. Finally, there is a limited understanding of how edge effects and habitat transitions influence rhizosphere communities, which is particularly relevant for fragmented landscapes where forest and grassland habitats are in close proximity [20]. Addressing these knowledge gaps is essential for predicting how ongoing land use changes affect plant–soil interactions, ecosystem functioning, and plant community dynamics in natural systems.

The primary objective of this study was to characterize the variations in the rhizosphere microbiota and metabolite profiles of naturally occurring *Barbarea vulgaris* populations grown across adjacent grasslands and forests, two different land uses. The specific objectives included (1) quantifying rhizosphere microbial community abundance and phenotypic structure dominance variation; (2) identifying and comparing rhizosphere metabolites by untargeted metabolomics; and (3) identifying key metabolic processes potentially influenced by land use. These integrated approaches aimed to provide insights into how contrasting land uses shape rhizosphere functioning and plant-microbiota-metabolite interactions in naturally growing *B. vulgaris* populations.

## 2. Materials and Methods

### 2.1. Field Trial

**Figure 1:**
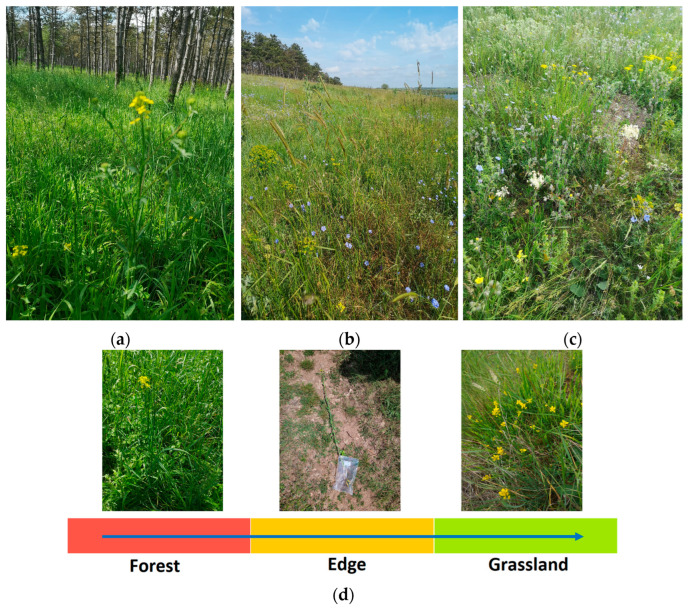
Field images with adjacent land uses where *Barbarea vulgaris* naturally grows. (**a**) Forest; (**b**) panoramic view of forest-grassland; (**c**) grassland; (**d**) schematic representation of the applied sampling transect.

Three adjacent habitat, forest, grassland, and edge (transitional zone), located near Limanu, Dobrogea, (43°48′44″ N, 28°29′52″ E) were selected for this study (Figure 1). The edge habitat represents the transitional zone between the forest and grassland, providing an intermediate environmental gradient for investigating rhizosphere biochemical networks. The first land use was an artificially planted black pine (*Pinus nigra Arn*.) forest with *Lolium perenne*, *Barbarea vulgaris*, *Clematis vitalba*, and *Bromus sterilis* as cover plants between trees. The herbaceous species present in the adjacent grassland (second land use) were *Barbarea vulgaris*, *Orlaya grandiflora*, *Euphorbia seguieriana*, *Euphorbia esula*, *Carduus nutans*, *Filipendula vulgaris*, *Verbascum phoeniceum*, *Allium rotundum*, *Linum perene*, *Adonis vernalis*, *Ornithogalum umbellatum*, *Salvia nemorosa*, *Salvia aethiopis*, *Potentilla recta*, *Onopordum tauricum*, *Thymus marschallianus*, *Adonis aestivalis*, and *Lolium perenne*. The climate of the region is warm-temperate continental, characterized by cold and dry winters and hot summers (Köppen–Geiger class Dfa). The mean annual temperature is approximately 12.5 °C, with a mean winter temperature (December–February) of approximately 2 °C and a mean summer temperature (June–August) of 25.5 °C. The annual precipitation is approximately 395 mm. The geology of the region comprises Sarmatian limestones covered by late Quaternary loess deposits, with soils primarily represented by Haplic and Luvic Chernozems and Phaeozems (IUSS WRB).

### 2.2. Barbarea vulgaris Rhizosphere Soil Sampling

The rhizosphere soils of mature *Barbarea vulgaris* were collected by shaking the roots and scraping the tightly adhering soils with sterile cotton swab after vegetable harvest. A total of 15 individual plants were used for rhizosphere soil sample collection from the two-land use transects (5 forest + 5 edge + 5 grassland, Figure 1d). Each sample constituted the total rhizosphere soil fraction obtained from an individual *Barbarea vulgaris* plant, representing the complete root-associated microbial and metabolite pool for that plant. To ensure sample independence, individual plants were selected with a minimum distance of 1.5 m between them. These soil samples were stored in a −20 °C mobile freezer until they arrived at the laboratory, after which the required amount for PLFA and metabolite analysis was moved to −80 °C. The soil physicochemical properties in terms of pH, water-extractable ions and soil organic matter were determined according to the methods described in Kovacs et al. [21]. Obtained data are presented in Supplementary Materials Table S1.

### 2.3. Rhizosphere Soil Microbial Phenotypic Structure Assessment

The soil microbiota living fraction phenotypic structure abundance was determined by phospholipid-derived fatty acid (PLFA) analysis following the method described in Kovacs et al. [22]. Briefly, lipids were extracted with modified Blight and Dyer reagents and fractionated on a silica column [23]. The phospholipid fraction was subjected to trans-esterification, and the resulting fatty acid methyl esters were quantified through GC-FID [22]. The MIDI Sherlock™ Microbial Identification System (Microbial ID, Newark, DE, USA) was used to assign individual PLFA biomarkers to functional bacterial (B) and fungal (F) guilds: Gram-positive (GP) bacteria, Gram-negative (GN) bacteria, aerobic bacteria, anaerobic bacteria, actinomycetes, arbuscular mycorrhizal fungi (AMF), ectomycorrhizal fungi (EMF) and saprotrophic fungi (SF). The molar sums of each guild were expressed as nmol g^−1^ dry soil, and dominance indices were derived as GP:GN, Aerobe:Anaerobe, F:B ([AMF + EMF + SF]/[GP + GN + Aerobe + Anaerobe + Actinomycetes]), Actinomycetes:PLFA, Bacteria:PLFA ([GP + GN + Aerobe + Anaerobe + Actinomycetes]/total PLFA), and Fungi: PLFA ([AMF + EMF + SF]/total PLFA). The total PLFAs represented the sum of all the determined PLFA biomarkers. Ratios > 0.5 represent the relative predominance of the numerator guild, providing a quantitative measure of shifts in community structure.

### 2.4. Mass Spectrometric Assessment of Untargeted Metabolites in Rhizosphere Soil

Rhizosphere soil untargeted metabolites were extracted from 1 g of lyophilized soil samples. The samples were homogenized in 750 µL of an ice-cooled 1:2 (*v*/*v*) chloroform/methanol mixture for 5 min. Next, 250 µL of ice-cooled chloroform was added, and the mixture was homogenized with sample–solvent mixtures. After 5 min, this step was repeated, with 250 µL of ice-cooled deionized water added to the mixture. The mixture was incubated for 30 min on ice, after which the homogenate was centrifuged at 2000 rpm for 15 min. Layer separation was performed on ice, where the upper layer was maintained and the lower layer was discarded. The obtained residual chloroform was subjected to re-extraction twice with 450 µL of ice-cooled 1:2 (*v*/*v*) chloroform/methanol, 150 µL of ice-cooled chloroform and 150 µL of ice-cooled deionized water. The resulting three upper layers were pooled together. The obtained approximately 1000 µL extracts were split into two approximately equal parts for mass spectrometric analysis as follows:

(a) GC–MS/MS analysis of untargeted metabolites—in that case, to the extract 100 µL of methoxyamine hydrochloride solution (0.02 g·mL^−1^ in pyridine) was added and allowed to react for 60 min at 30 °C. Next, 100 µL of MSTFA was added, and the mixture was incubated at 50 °C for 30 min. After incubation, 2 µL was injected into a Thermo Finningan gas chromatograph linked to a triple quadruple mass spectrometer (Trace 1310 GC-TSQ 9000 MS) equipped with an SSL injector port set at 250 °C. The metabolites were separated through an HP-5MS capillary column (30 m × 0.25 mm × 0.25 µm; Hewlett Packard, Palo Alto, CA, USA). Helium was used as the carrier gas at a flow rate of 1 mL·min^−1^. The oven temperature program started at 40 °C for 7 min^−1^, followed by a ramp of 5 °C·min^−1^ to 285 °C, held at this final temperature for 7 min. The solvent delay time was set at 3.5 min. The ion source was set at 230 °C and operated in electron ionization mode (EI) at 70 eV. The mass spectrometer was operated in full scan mode with an ion scanning range of 50–550 *m*/*z*. Primary data analysis was conducted through Xcalibur 4.0 software, followed by MS-DIAL version 4.9 software according to the procedures described by Lai et al. [24] and Kovacs et al. [7]. Process blanks (extraction solvent without soil sample) were prepared and analyzed alongside samples to identify background contamination, and features detected in blanks at >20% of the average intensity were excluded from downstream analysis. Further, the criterion for compound identification was a mass-spectrum matching score of ≥80%. Each sample’s total ion chromatogram (TIC) was used for peak-area integration. The results were expressed as a percentage of the relative peak area of a peak for each soil sample, which was calculated by dividing the peak area by the total peak area of all identified peaks in each chromatogram.
(b) MALDI TOF/TOF MS analysis of untargeted metabolites—In that case, the remaining part of the extract was dried under a gentle stream of N2, after which it was reconstituted in a 25 µL mixture of 0.05% TFA, water and 2% ammonium hydroxide. Next, 2.5 µL of reconstituted sample was mixed with 2.5 µL of 9-aminoacridine (9-AA matrix solution of 10 mg·mL^−1^ in 0.1% TFA in acetone). One microliter of this obtained sample mixture was pipetted onto a MALDI-TOF target (MTP 384 polished steel target Bruker Daltonics, Bremen, Germany) and allowed to air dry at room temperature. Mass spectra were acquired with a MALDI TOF/TOF IMS analyser (Autoflex maX, Bruker Daltonics, Bremen, Germany) equipped with a Smartbeam-II laser (Nd:Yag—355 nm). Prior to analysis, calibration was performed in linear mode using a standard mixture of metabolites (lactate, succinate, malate, AMP, ATP, and CoA). The instrument was operated in linear negative ion mode, which provides enhanced sensitivity and broader mass range coverage for low molecular weight metabolites while minimizing fragmentation of labile compounds. For acquisition, the laser intensity was set to 35%, and the laser frequency was set to 500 Hz. Approximately 2000 laser shots were totaled per raster position. Data analysis was performed by Bruker data management software flexAnalysis version 3.4 with the centroid peak detection algorithm and the baseline subtraction TopHat. The R-MetaboList 2 and rMSIfragment tools were subsequently used for metabolite annotation according to the procedures of Baquer et al. [25] and Peris-Diaz et al. [26].

### 2.5. Statistical Analysis

All the statistical analyses were performed with R statistical software (v 4.3.3, R Core Team). Prior to statistical analysis, data normality and homogeneity of variance were assessed with Shapiro–Wilk and Levene’s tests, respectively. Microbial community structure differentiation across land use types was assessed by permutational multivariate analysis of variance (PERMANOVA) implemented in the vegan package, with Bray–Curtis dissimilarity matrices calculated by the vegdist() function. Microbial phenotype ratios were compared by one-way ANOVA (aov() function) followed by Tukey’s honestly significant difference (HSD) post hoc test for pairwise comparisons. The ordination analyses included nonmetric multidimensional scaling (NMDS) using metaMDS() and principal coordinate analysis (PCoA) with cmdscale() functions with Bray–Curtis dissimilarity matrices implemented through the vegan package. Microbial community phenotypic relationships were analysed using distance-based approaches. For unweighted analysis, Hellinger-transformed data were used with Euclidian distance metrics. For weighted analysis, Bray–Curtis dissimilarity matrices were calculated using the vegdist() function from the vegan package. Hierarchical clustering was performed using the hclust() function with Ward.D2 linkage method and Euclidean distance matrices, calculated by dist() function for microbiome–metabolome correlation analysis. Metabolomic data preprocessing included log_2_ fold change transformation (log2() function) for relative abundance calculations. Metabolite identification was performed against the KEGG database by the KEGGREST package. Pathway enrichment analysis was conducted through metabolomics pathway analysis tools to identify significantly affected metabolic pathways, incorporating both statistical significance and topological impact measures through hypergeometric tests and pathway topology analysis. Co-inertia analysis was performed with the ade4 package with the coinertia() function to quantify the coupling strength between the microbiota, metabolite profile, and soil property datasets, with RV coefficients calculated using the RV.rtest() function to assess correlation strength and statistical significance through Monte Carlo permutation tests. Correlation analyses between microbial phenotypes and metabolite concentrations were performed with the cor() function from the stats package, with high-magnitude correlations defined as |r| > 0.8. Statistical significance was set at α = 0.05, with multiple significance levels reported: * *p* < 0.05, ** *p* < 0.01, *** *p* < 0.001. Multiple testing correction was applied using the Benjamini–Hochberg false discovery rate (FDR) method implemented with the p.adjust() function when needed. All multivariate analyses utilized corresponding permutation-based significance testing procedures to account for the nonindependence of the ecological data. Data visualization was performed using base R graphics and the ggplot2 package for ordination plots and correlation matrices.

## 3. Results

### 3.1. Microbiome Composition of the Rhizosphere Soil of Barbarea vulgaris Growing Under Different Land Uses

**Figure 2:**
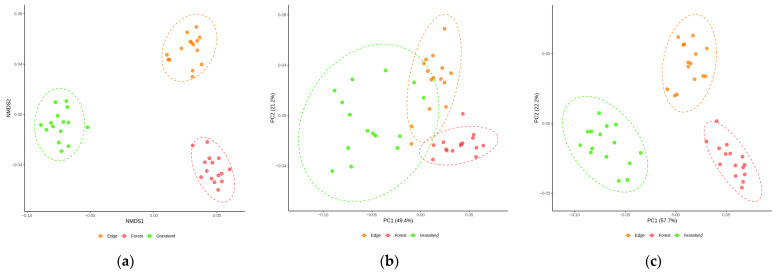
Differences in microbial phenotypic composition in the rhizosphere soil samples using NMDS and PCoA: (**a**) NMDS by the weighted UniFrac (WUF) metric; (**b**) PCoA with WUF; and (**c**) unweighted UniFrac (UUF). Colours indicate land use types: forest (pink), grassland (green), and edge (orange).

PLFA analysis of *Barbarea vulgaris* rhizosphere soil revealed microbiota abundances ranging from 147.1 to 191.8 nmol·g^−1^, with bacterial biomarkers in the 134.1–177.9 nmol·g^−1^ domain and fungal biomarkers in the 10.3–17.4 nmol·g^−1^ domain. The microbial community structure across the forest-edge-grassland gradient was evaluated using PLFA dominance ratios (Table 1) and multivariate ordination analysis (Figure 2). Among the five calculated dominance ratios (Table 1), only G+/G− showed significant habitat differentiation (F_2,12_ = 26.661, *p* < 0.001). The forest habitat displayed the highest G+/G− ratio, followed by the edge and grassland habitats, with Tukey’s HSD post hoc analysis confirming significant pairwise differences across all land use comparisons (Table 1). This gradient indicates a shift from Gram-positive-dominated communities in the more stable forest environment to Gram-negative-dominated communities in the more disturbed grassland habitat. The remaining four ratios showed no significant land use effects.

Multivariate ordination analysis complemented the univariate ratio assessments and revealed distinct land-use-specific clustering patterns. NMDS ordination by Bray–Curtis dissimilarity (stress = 0.0618) demonstrated good two-dimensional separation of the PLFA-derived community structure (Figure 2a). The rhizosphere microbiome of *Barbarea vulgaris* growing in grasslands formed a tight cluster, indicating low within-land use variability. In contrast, the forest rhizosphere soil microbial communities occupied a distinct region of the NMDS ordination and were clearly separated from the grassland assemblage along the NMDS1 axis. The edge showed intermediate positioning with greater dispersion. PERMANOVA confirmed significant land use differentiation (R^2^ = 0.61, F = 33.03, *p* < 0.001), with the greatest dissimilarity between the grassland-forest (R^2^ = 0.62, *p* < 0.001) and moderate differentiation for the grassland-edge and edge forest comparisons (R^2^ = 0.48 each, *p* < 0.001). Principal coordinate analysis (PCoA) based on Bray–Curtis dissimilarity explained 70.6% of the variance across PC1-PC2 (49.4% and 21.2%, respectively; R^2^ = 0.611, *p* = 0.001), emphasizing abundance-weighted differences (Figure 2b). The forest communities formed a distinct cluster, indicating specialized high-abundance phenotypes, whereas the grassland samples formed compact clusters, suggesting homogeneous dominant species composition. The edge samples occupied intermediate positions with moderate dispersion. Unweighted analysis (Hellinger-transformed Euclidean distances) captured 79.9% of the variance (PC1: 57.7%, PC2: 22.2%; R^2^ = 0.734, *p* = 0.001), with a focus on presence/absence patterns (Figure 2c).

### 3.2. Untargeted Metabolite Analysis of Rhizosphere Soil of Barbarea vulgaris Grown Under Different Land Uses

**Figure 3:**
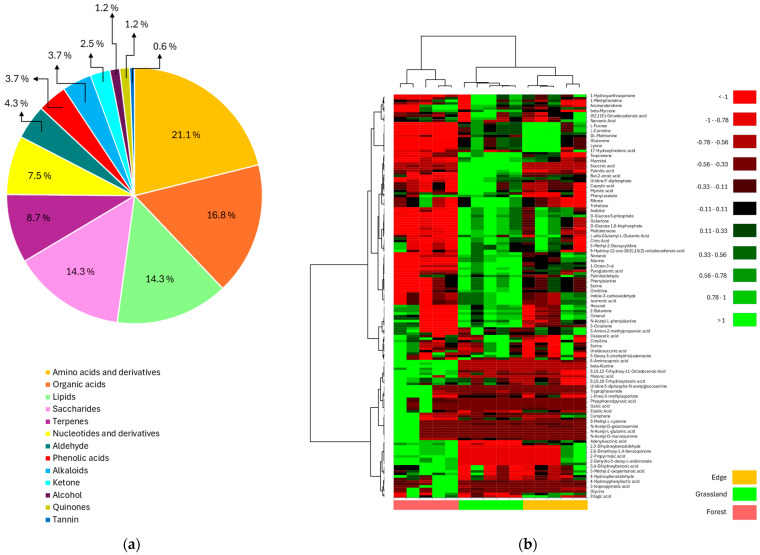
*Barbarea vulgaris* rhizosphere metabolites: (**a**) major metabolite group identified in the *Barbarea vulgaris* rhizosphere soil; (**b**) clustered heatmap of rhizosphere soil metabolite variation across *Barbarea vulgaris* grown under different land uses. Colours indicate land use types: forest (pink), grassland (green), and edge (orange).

Mass spectrometric analysis of *Barbarea vulgaris* rhizosphere soil samples detected 248 metabolites, of which 161 could be annotated against the Kyoto Encyclopedia of Genes and Genomes database of molecules (https://www.genome.jp/ accessed on 10 June 2025). These metabolites were subsequently considered next for metabolite profiling and pathway analysis. The pie chart (Figure 3a) displays their distribution by chemical class after grouping them into 13 major categories based of their chemical properties. Amino acids and their derivatives were the dominant fraction at 21.1%, followed by organic acids at 16.8%. Lipids and saccharides each represent 14.3% of the total metabolite composition. Terpenes account for 8.7%, whereas nucleotides and their derivatives constitute 7.5%. Minor constituents include aldehydes (4.3%), phenolic acids (3.7%), alkaloids (3.7%), ketones (2.5%), alcohols (1.2%), quinones (1.2%), and tannins (0.6%). The data revealed diverse metabolic profiles, with nitrogen-containing compounds being most prevalent, followed by carbon-based organic acids and structural lipids.

The clustered heatmap (Figure 3b) displays metabolomic profiles of rhizosphere soil from *Barbarea vulgaris* across three land use types: edge (yellow), grassland (green), and forest (red). Rows represent individual metabolites and column represent samples, with hierarchical clustering performed using Euclidian distance and Ward’s linkage. The color scale represents log_2_-fold changes, with values > 1 indicating upregulation and <−1 indicating downregulation relative to the mean. Distinct metabolic clusters emerged, including groups enriched in amino acids, organic acids, and secondary metabolites, including carboxylic acids, phenolic compounds, and nitrogen-containing metabolites. The clustering patterns revealed land-use-specific metabolic signatures, with certain metabolites consistently elevated or depleted in specific habitats. The dendrogram topology demonstrated metabolite co-occurrence patterns and clear metabolomic differentiation between the three land use types.

### 3.3. Metabolic Pathway Analysis of the Barbarea vulgaris Rhizosphere Soil Across Land Use Types

**Figure 4:**
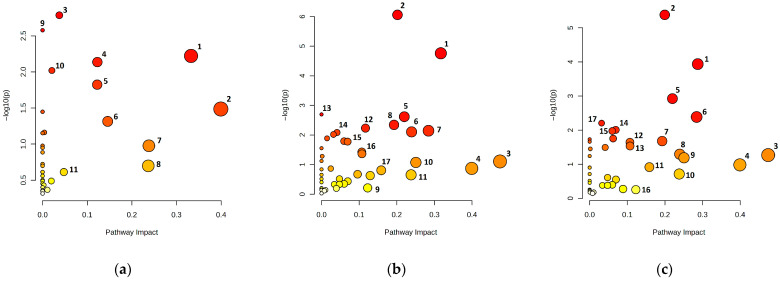
Variation in the enriched metabolomic pathways in the rhizosphere soils of *Barbarea vulgaris* grown under different land uses. (**a**) Grassland vs. edge; (**b**) edge vs. forest; (**c**) forest vs. grassland. Each number corresponding enriched metabolomic pathway is listed in Supplementary material Table S2.

Pairwise comparisons of rhizosphere metabolomic profiles revealed distinct pathway enrichment patterns across land uses (Figure 4 and Supplementary Materials Table S2). Pathway enrichment analysis of the rhizosphere soil of *Barbarea vulgaris* growing on grasslands and edges revealed significant metabolic differences (Figure 4a, Table S2). Pyrimidine metabolism showed the greatest statistical significance, followed by nitrogen metabolism. Nitrogen metabolism presented zero topological impact. Alanine, aspartate, and glutamate metabolism demonstrated both high significance and substantial topological impact. Beta-alanine metabolism had an impact value of 0.4, whereas glycolysis and gluconeogenesis had impacts of 0.15. Moderate enrichment was observed in the lysine degradation and arginine biosynthesis pathways. Additional pathway perturbations were detected in phenylalanine and glycerolipid metabolism. The edge vs. forest comparison revealed pathway perturbations in central carbon and nitrogen metabolism (Figure 4b, Table S2). Alanine, aspartate, and glutamate metabolism presented the strongest statistical significance, with moderate topological impact. TCA and glycolysis/gluconeogenesis had impacts of 0.32 and 0.28, respectively. Enrichment was observed in pyruvate metabolism and the glyoxylate and dicarboxylate pathways. Changes in arginine biosynthesis and phenylalanine metabolism were detected. Moderate effects were detected in the nucleotide metabolism (pyrimidine) and carbohydrate processing (amino sugar, galactose) pathways. Ascrobate/aldarate pathway enrichment was also observed. The forest vs. grassland comparison revealed pathway alterations in central carbon and nitrogen metabolism (Figure 4c, Table S2). Alanine, aspartate, and glutamate metabolism exhibited the strongest enrichment, with a topological impact of 0.2. The TCA cycle had an impact of 0.29, followed by the glycolysis and gluconeogenesis metabolic pathways, with an impact of 0.28. The glyoxylate and dicarboxylate metabolic pathway presented an impact of 0.22. Pyruvate metabolism and arginine biosynthesis had impacts ranging from 0.19 to 0.24, whereas glycine, serine and threonine metabolism presented the highest topological impact of 0.47, with lower statistical significance. Changes were observed in carbohydrate-processing pathways (starch/sucrose and pentose phosphate pathways) and sulfur-mediated one-carbon metabolism.

### 3.4. Correlations Between the Microbiota and Metabolites of Barbarea vulgaris Rhizosphere Soil

**Figure 5:**
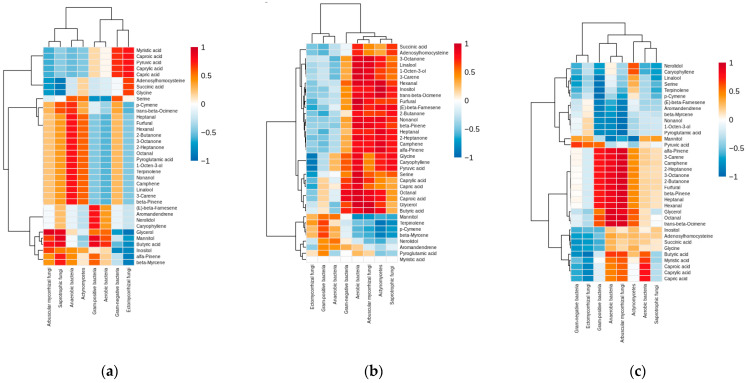
Correlation analyses of the soil *Barbarea vulgaris* rhizosphere soil microbiome and metabolome. Hierarchical clustering heatmap of the soil metabolites based on the microbiota phenotypic structure in different land uses: (**a**) grassland; (**b**) edge; (**c**) forest.

Microbiome–metabolome correlation analysis revealed distinct patterns across the three land use types examined (Figure 5).

Ward.D2 hierarchical clustering with Euclidean distance metrics was applied consistently across all analyses. Metabolite clustering patterns were consistent across land uses, with compounds grouped into three primary chemical classes. The volatile organic compounds, including monoterpenes and sesquiterpenes, formed distinct clusters in all the dendrograms. Carboxylic acids and other organic acids clustered separately from amino acid derivatives and fatty acid metabolites. Biosynthetically related compounds showed coregulation patterns, which was particularly evident in the grassland dendrogram (Figure 5a). Microbial community clustering separated bacterial and fungal taxa across all land uses. The bacterial groups included Gram-positive bacteria, Gram-negative bacteria, and actinomycetes, with aerobic and anaerobic bacteria forming distinct dendrogram branches. Fungal communities clustered into saprotrophic fungi, ectomycorrhizal fungi, and arbuscular mycorrhizal fungi, with clear phenotypic separation observed in forests (Figure 5c). Grassland (Figure 5a) correlations revealed positive associations between Gram-negative bacteria and volatile organic compounds. Actinomycetes was negatively correlated with specific organic acids. The grassland correlation matrix revealed the most structured relationships, with several perfect correlations (r = ±1.00) observed between the microbial phenotypes and metabolite concentrations. Edge (Figure 5b) correlations revealed strong positive associations between Gram-negative bacteria and monoterpene compounds. Negative correlations were observed between actinomycetes and fatty acid metabolites. The correlation matrix exhibited several near-perfect positive and negative coefficients, indicating highly structured biochemical interactions. The forest (Figure 5c) correlations revealed positive associations between saprotrophic fungi and specific terpene compounds. Gram-positive bacteria were negatively correlated with certain organic acids. Compared with bacterial associations, fungal–metabolite relationships presented particularly strong correlation coefficients. The dendrogram structures were consistent across land uses, with metabolite clustering reflecting chemical similarity and microbial clustering reflecting microbial phenotypic and functional relationships. High-magnitude correlations (|r| > 0.8) were present throughout all the correlation networks, indicating strong microbiome–metabolome coupling in *Barbarea vulgaris* rhizosphere soils.

### 3.5. Co-Inertia Analysis of Land Use and the Microbiome-Metabolome of Barbarea vulgaris Rhizosphere Soil

Co-inertia analysis was employed to quantify the co-structure and coupling strength between different datasets (microbiota, metabolites, and soil properties) within *Barberea vulgaris* rhizosphere communities across varying land use types.

On the basis of co-inertia analysis (Table 2), this study examined pairwise relationships between microbiota, metabolites, and soil properties across *Barbarea vulgaris* rhizosphere soil grown in different uses. In the case of *Barbarea vulgaris* growing in forest, the rhizosphere soil presented the strongest metabolite–soil property coupling (RV = 0.91, 81.2% first axis variance, ***), indicating exceptionally tight coordination between the soil chemistry and metabolite profiles. In contrast, the microbiota–soil relationships were moderately strong (RV = 0.68, 58.2% variance, *), suggesting that while microbial communities respond to soil conditions, this relationship is less pronounced than metabolite–soil coupling. For *Barbarea vulgaris* growing at the edge, the rhizosphere soil presented distinct patterns, with the highest microbiota–soil property co-structure (RV = 0.81, 79.2% variance, **). These findings suggest that microbial communities in transitional zones strongly reflect underlying soil characteristics. Additionally, metabolite–soil coupling remained substantial (RV = 0.74, 89.2% variance, *), indicating that edge environments maintain strong biochemical–edaphic relationships. For grasslands, the rhizosphere soil presented the most balanced yet weakest relationships across all comparisons, with RV values consistently ranging from 0.5 to 0.6. The first axis variance spanned 48.3–71.5%, reflecting more moderate environmental structuring than the other land uses did. Notably, the microbiota–metabolite relationships remained consistently moderate across all land uses (RV = 0.5–0.72, 62.8–84.9% variance, *), indicating stable functional connections between microbial communities and metabolite production. This consistency suggests that fundamental biochemical processes persist across different environmental contexts. The high first axis variance values (48.3–89.2%) across all analyses indicate that strong primary environmental gradients structure these multivariate relationships. Edge environments displayed particularly integrated co-structure across all three datasets.

## 4. Discussion

### 4.1. Land-Use-Driven Microbiome–Metabolome Coupling in the Barbarea vulgaris Rhizosphere

The present study revealed that land use influences the microbial community structure in *Barbarea vulgaris* populations rhizosphere, with distinct phenotypic structures and abundances observed across samples from forests, edges, and grasslands (Figure 2). The differences in G+/G− dominance between forest and grassland land uses (*p* < 0.001, Table 1) could be attributed to differences in carbon availability and soil chemistry (Supplementary Material, Table S1). Gram-positive bacteria are generally more efficient at utilizing complex organic compounds and are better adapted to nutrient-limited conditions characteristic of forest soils [27]. Samaniego et al. [28] explained that organic matter has a slower decomposition rate because of the higher C:N ratios and the presence of recalcitrant compounds such as lignin and tannins. Conversely, bacterial dominance in grassland soils likely reflects the more labile nature of root exudates [29] and organic inputs [30] in these systems, which favour fast-growing Gram-negative bacteria capable of rapid nutrient uptake and metabolism, as demonstrated by Samaniego et al. [28] and Maciel-Rodriguez et al. [31]. Such shifts in microbial community composition could have significant implications for plant performance, as Gram-negative bacteria in grasslands facilitate rapid nutrient mobilization and cycling that supports faster growth rates, while Gram-positive bacteria in forests enhance plant access to recalcitrant organic nutrients under resource-limited conditions, thereby improving stress tolerance [28,29]. The intermediate microbial signatures observed in edge environments support the concept of ecotones as transitional zones with unique ecological properties [32]. These edge effects have been extensively documented in landscape ecology [33,34], where boundary zones between contrasting habitats often exhibit distinct community assemblages that reflect the influence of both adjacent ecosystems [35]. The microbial community structure in edge soils suggests a gradient of environmental conditions that promotes functional diversity intermediate between forest and grassland extremes, potentially providing *Barbarea vulgaris* with metabolic flexibility to cope with fluctuating environmental conditions.

The detection of 248 metabolites, 161 of which were successfully mapped to KEGG pathways, provides insight into the biochemical complexity of *B. vulgaris* rhizosphere environments. The predominance of amino acids and derivatives and organic acids (Figure 3a) among the detected metabolites reflects the central role of nitrogen and carbon metabolism in rhizosphere biochemistry. On the basis of the findings of Canarini et al. [36] and Wankhade et al. [3], this metabolite composition is consistent with the known functions of root exudates, which serve as signalling molecules, antimicrobial compounds, and nutrient sources for rhizosphere microorganisms. The distinct metabolite profiles observed across land use types indicate that environmental conditions influence the biochemical landscape of the rhizosphere, with potential consequences for plant–microbe interactions and plant fitness. Forest soils were enriched in metabolites associated with complex organic matter decomposition and stress response pathways (Figure 3b; Supplementary Material Table S2), reflecting the more challenging environmental conditions and slower nutrient cycling characteristics of these ecosystems [28]. The presence of specialized metabolites in forest rhizospheres may represent adaptive responses to nutrient limitation, pathogen pressure, or allelopathic interactions with competing vegetation [37], enabling *Barbarea vulgaris* to maintain growth and defence capabilities under resource-constrained conditions. Grassland environments enriched metabolites related to rapid nutrient cycling and primary metabolism (Supplementary Material Table S2), suggesting enhanced capacity for fast growth and resource acquisition which is consistent with the more dynamic and nutrient-rich conditions typical of these systems [29]. The higher abundance of amino acids and simple organic compounds in the grassland soils (Figure 3b) likely reflects increased microbial activity and faster turnover of organic matter, facilitating more efficient nutrient mobilization and plant uptake [30]. Together, these patterns indicate that land use modifies the availability and quality of rhizosphere carbon and nitrogen pools in ways that directly shape plant functional trade-offs. In forests, higher representation of TCA cycle intermediates and aromatic-derived compounds is consistent with a more resource-conservative strategy that supports nutrient scavenging (via organic acid-mediated mobilization), oxidative stress mitigation, and defence priming. In grasslands, enrichment of amino acids and glycolytic intermediates aligns with increased nitrogen cycling and readily utilizable carbon that can promote faster microbial growth and, consequently, higher mineralization and nutrient release, potentially enhancing plant growth under favourable conditions.

Pathway enrichment analysis (Figure 4) revealed coordinated metabolic reprogramming across land use gradients, with particularly pronounced effects on central carbon and nitrogen metabolism. The comparison between grassland and edge environments (Figure 4a, Supplementary Material Table S2) revealed perturbations in pyrimidine metabolism (−log10(p) = 2.79) and nitrogen metabolism (−log10(p) = 2.58), although the latter had minimal topological impact, suggesting broad but peripheral metabolite adjustments rather than core network disruption. The substantial enrichment and high impact of alanine, aspartate and glutamate metabolism (−log10(p) = 2.22, im-pact 0.33) indicate central network perturbation in amino acid interconversions and nitrogen shuttling, which is consistent with the fundamental role of these pathways in plant–microbe nitrogen exchange [38]. The concurrent perturbation of beta-alanine metabolism (impact of 0.40) and glycolysis/gluconeogenesis (impact of 0.15) suggests coordinated adjustments in carbon flux and osmo-protectant synthesis, reflecting adaptive responses to varying osmotic and nutritional stresses between these environments according to Zhang et al. [39]. The edge versus forest comparison (Figure 4b, Supplementary Material Table S2) revealed more pronounced metabolic restructuring, with alanine, aspartate and glutamate metabolism showing the strongest significance (−log10(p) ≈ 6.06), indicating intensified transamination flux at the forest-edge interface. This pattern aligns with previous observations that ecotone environments often exhibit heightened metabolic activity due to resource gradients and increased plant–microbe interactions [32,40]. The concurrent perturbation of the TCA cycle (−log10(p) ≈ 4.76, impact ≈ 0.32) and glycolysis/gluconeogenesis (impact ≈ 0.28) suggests coordinated reprogramming of energy metabolism, potentially reflecting altered carbon availability or redox conditions in edge rhizospheres (Supplementary Material, Table S1). The enrichment of pyruvate metabolism and glyoxylate/dicarboxylate pathways further supports the remodelling of anaplerotic routes and C2 assimilation [41], which is consistent with the metabolic flexibility required in transitional environments where resource availability fluctuates [42]. The forest versus grassland comparison (Figure 4c, Supplementary Material Table S2) revealed the most comprehensive metabolic restructuring, with alanine, aspartate and glutamate metabolism again showing strong enrichment (−log10(p) = 5.38, impact = 0.20), reinforcing the central role of amino acid metabolism in habitat-specific adaptations [38]. The concurrent perturbation of the TCA cycle (−log10(p) = 3.94, impact = 0.29) and glycolysis/gluconeogenesis (−log10(p) = 2.39, impact = 0.28) indicates rewiring of energy metabolism between these contrasting environments. This metabolic reprogramming likely reflects the transition from the complex, recalcitrant organic matter characteristic of forest soils [28] to the more labile, rapidly cycling organic inputs typical of grassland systems [29]. The elevated impact of glyoxylate/dicarboxylate metabolism (impact = 0.22) implies enhanced anaplerotic capacity and C2 assimilation efficiency [42]. Notably, glycine, serine and threonine metabolism had the greatest topological impact (0.47) in the forest-grassland comparison, despite its lower statistical significance, indicating targeted perturbations at critical network nodes. This pattern suggests that specific metabolic bottlenecks, rather than broad pathway-level changes, may drive functional differentiation between these land uses environments. The involvement of these amino acids in one-carbon metabolism and methylation reactions implies altered epigenetic regulation and stress response mechanisms across habitats [43]. The enrichment of carbohydrate-processing pathways, including starch/sucrose and pentose phosphate metabolism, indicates habitat-specific adjustments in osmolyte turnover [44] and NADPH supply [45]. The pentose phosphate pathway is particularly important for generating the reducing power required for biosynthetic processes [46] and antioxidant defence [47], suggesting enhanced oxidative stress management. The concurrent perturbation of the sulfur and one-carbon metabolism pathways points to altered methylation capacity [48] and sulfur assimilation across land uses [49], processes that are crucial for protein synthesis and stress tolerance [50].

The significant correlations observed between the microbial community structure and metabolite profiles (Figure 5) demonstrate the existence of tightly coupled biochemical networks in the rhizosphere. This microbiome–metabolome coupling suggests that microbial communities and metabolite pools co-evolve to optimize ecosystem functioning under specific environmental conditions. Hierarchical clustering analysis revealed distinct correlation patterns between microbial phenotypes and metabolite classes, indicating that specific microbial groups are associated with particular biochemical functions. Fungal biomass was positively correlated with aromatic compounds and complex organic acids, which is consistent with the role of fungi in decomposing lignin and other recalcitrant organic matter [28]. Conversely, bacterial biomass is positively correlated with amino acids and simple organic compounds, reflecting bacterial preferences for consuming these more labile substrates [30]. Furthermore, the bacterial communities correlated with metabolites involved in the stress response and secondary metabolism. While these correlations may reflect either microbial production or utilization of defensive compounds, the association is consistent with Gram-positive bacteria playing important roles in secondary metabolites dynamics, as evidenced by Lv et al. [51]. This relationship may reflect the generally greater stress tolerance of Gram-positive bacteria and their ability to survive in nutrient-limited conditions where secondary metabolite production or transformation is advantageous [27]. The stronger coupling observed in forest environments (Figure 5c) than in grassland rhizosphere soils (Figure 5a) may reflect the more stable environmental conditions and longer-established plant–microbe associations characteristic of forest ecosystems. This enhanced coupling could contribute to greater ecosystem stability and resilience to environmental perturbations, as tightly integrated networks are often more resistant to disruption [52].

Co-inertia analysis quantifying the coupling between microbiota, metabolites, and soil properties provides a framework for understanding ecosystem-level interactions. The varying coupling strength across land uses suggests that the environmental context fundamentally shapes the organization and functioning of rhizosphere biochemical networks. The forest environments presented the highest coupling coefficients, indicating strong coordination between the microbial community structure, metabolite profiles, and soil chemical properties, which is consistent with the complex, multi-trophic interactions characteristic of mature forest ecosystems [53].

### 4.2. Ecological Implications

The differential organization of rhizosphere biochemical networks across land use types has profound implications for ecosystem functioning and plant community dynamics [54]. While our study focuses on *Barbarea vulgaris* as a model system, this species can serve as an ecological relevant indicator for understanding broader rhizosphere processes across forest-grassland gradient. *Barbarea vulgaris* is widely distributed across temperate ecosystems and present habitat generalist characteristics [55], making it particularly suitable for comparative studies across land use types. By focusing on a single species present across all land use types, our design effectively controlled for species-specific rhizosphere chemistry allowing us to attribute observed biochemical reorganization to habitat-driven processes rather than plant taxonomic variations. Moreover, the metabolic pathways most responsive to land use change—nitrogen metabolism, amino acid biosynthesis, and carbon cycling—represents phylogenetically conserved processes central to plant-microbe nutrient exchange across taxa, suggesting that our findings capture general mechanisms of rhizosphere reorganization rather than species-specific responses. Although PLFA analysis provides phenotypic rather than taxonomic characterization of microbial communities, this approach offers advantages for understanding functional relationships in rhizosphere systems. PLFA profiles directly reflect the living microbial biomass and physiological status of communities, capturing functional shifts in microbial dominance that are mechanistically linked to metabolic processes. The significant microbiome-metabolome coupling we observed (RV = 0.91, Table 2) show that PLFA-based community characterization is sufficiently sensitive to detect functionally relevant microbial variation. This phenotypic approach may actually be more appropriate for metabolomic integration studies than high-resolution taxonomic methods, as PLFA biomarkers directly reflect membrane composition changes associated with metabolic activity and environmental adaptation. Furthermore, the alignment of our results with previous studies that involved high-throughput sequencing approaches [56] confirms the PLFA-based phenotypic profiling reliably captures the functional microbial shifts that drive ecosystem-level processes, despite differences in methodological resolution. The pronounced microbiome–metabolome coupling observed in forest environments (RV = 0.91, Table 2) suggests that mature ecosystems develop highly integrated biochemical networks that increase resource use efficiency and ecosystem stability [52]. This tight coupling likely contributes to the resilience of forest ecosystems by creating redundant pathways for nutrient cycling and stress response [53], which is consistent with the insurance hypothesis of biodiversity–ecosystem functioning relationships [57]. The land-use-specific metabolic pathway enrichment patterns indicate fundamental differences in biogeochemical cycling across land uses. The enhanced nitrogen metabolism and amino acid interconversion pathways in forest–grassland transitions suggest that land use change triggers adaptive metabolic reprogramming that may influence plant competitive abilities and community assembly processes [38,40]. The enrichment of aromatic amino acid biosynthesis pathways in forest environments implies increased investment in defensive secondary metabolites [58], potentially affecting plant–herbivore interactions and allelopathic relationships within plant communities [41]. The intermediate coupling strength observed in edge environments (RV = 0.81 for microbiota–soil relationships) highlights the unique ecological role of ecotones in maintaining landscape-scale functional diversity [32]. These transitional zones may serve as reservoirs of metabolic and microbial diversity, facilitating species migration and adaptation under changing environmental conditions. The greater variability in microbial community structure observed in edge habitats suggests that these environments may be particularly important for maintaining regional biodiversity and ecosystem resilience. The distinct metabolic signatures associated with each land use type have implications for plant adaptation and evolution [59]. The coordinated shifts in carbon and nitrogen metabolism pathways suggest that rhizosphere biochemistry co-evolves with habitat conditions, potentially driving local adaptation in plant populations, as stated by Li et al. [38]. This metabolic specialization may influence plant performance across environmental gradients and affect species responses to climate change and habitat fragmentation [40,43]. While our study examined a single plant species, the metabolic pathways we identified—including central carbon metabolism, nitrogen assimilation, and secondary metabolite biosynthesis—are highly conserved across plant lineages, suggesting that the land-use-dependent reorganization patterns we observed likely operate across diverse plant communities. Future multi-species comparisons would be required for quantifying the extent to which species-specific rhizosphere chemistry modulates these general patterns. The significant correlations between microbial community structure and metabolite profiles across all land uses indicate that alterations in microbial community dominance through land use change or other anthropogenic factors could cascade through metabolic networks, potentially compromising ecosystem functions such as nutrient cycling, carbon sequestration, and plant productivity. The functional group-level resolution provided by PLFA analysis is relevant for predicting these ecosystem-scale responses, as shifts in broad microbial guilds (e.g., bacterial vs. fungal dominance) often have stronger effects on biogeochemical processes than changes in fine-scale taxonomic composition [59]. Our findings thus provide a functional framework for understanding how land use intensification affects rhizosphere processes, with implications extending beyond the specific study system to inform strategies across temperate forest-grassland landscapes. Understanding these biochemical network dependencies is crucial for predicting ecosystem responses to environmental change and developing effective conservation strategies that maintain functional rhizosphere processes across heterogeneous landscapes.

### 4.3. Current Research Limitations

While this study provides insight into the effects of land use on rhizosphere biochemical networks through integrated microbial–metabolomic analysis, methodological constraints warrant consideration. The cross-sectional sampling design, although enabling comprehensive spatial comparisons across forest–grassland gradients does not capture temporal dynamics that can vary seasonally and inter-annually. Rhizosphere processes are known to fluctuate with plant development stage, meteorological conditions, soil moisture, and phenology, all of which can restructure microbial communities and metabolite profiles over time. Consequently, the present findings reflect a temporal snapshot rather than the full seasonal trajectory of microbiome-metabolome coupling. Future longitudinal designs that repeatedly sample the same individuals and sites across key phenophases (e.g., early growth, flowering, senescence) and seasonal transitions (e.g., wet-dry cycles, temperature shifts) are needed to quantify within-site temporal variance, test for time-lagged microbiome-metabolome responses, and differentiate persistent habitat signals from transient seasonal effects. Incorporating mixed-effects models with random effects for plant identity and site, along with repeated measures of environmental covariate, would provide stronger inference on temporal stability versus plasticity in these networks. Our focus on *Barbarea vulgaris*, while allowing controlled comparisons of habitat-specific responses, limits its generalizability to broader plant community interactions, given species-specific rhizosphere chemistry variations. The PLFA-based microbial characterization successfully quantified phenotypic abundance patterns and dominance variations, revealing significant land use effects on bacterial communities, but lacked the taxonomic resolution that high-throughput sequencing could provide for identifying specific microbial taxa driving metabolic processes. Our untargeted metabolomics approach effectively detected 248 metabolites with robust pathway mapping (161 metabolites to KEGG), suggesting coordinated metabolic reprogramming across habitats. However, this likely represents a fraction of the total rhizosphere metabolome, with annotation gaps potentially limiting the functional interpretation of unmapped compounds. The individual plant-scale sampling strategy, while capturing fine-scale rhizosphere processes, may not reflect landscape-level dynamics or spatial autocorrelation effects. Despite these constraints, our co-inertia analysis revealed strong microbiome–metabolome coupling (RV = 0.91 in forests), providing evidence for habitat-specific biochemical integration. The correlative nature of our associations, although precluding potential causal inference, establishes a foundation for future experimental manipulation studies to validate the mechanistic relationships between the microbial community structure and metabolite production pathways.

## 5. Conclusions

This study provides quantitative evidence for the land-use-driven differentiation of rhizosphere biochemical networks in *Barbarea vulgaris* populations. PLFA-based microbial community analysis revealed land-use-specific signatures, especially in case of bacterial community. The untargeted mass spectrometric metabolomics approach identified metabolites that belong to 13 major groups on the basis of their chemical properties, with pathway enrichment analysis suggesting coordinated reprogramming of central carbon and nitrogen metabolism across land use gradients. The microbiome–metabolome coupling analysis revealed distinct correlation patterns between microbial phenotypes and metabolite classes, with forest environments showing the strongest biochemical network integration. Edge habitats presented intermediate microbial community structure and metabolite profiles, supporting their role as transitional zones with unique biochemical properties. Pathway-level analysis revealed perturbations in amino acid metabolism, particularly alanine, aspartate and glutamate interconversion, and energy metabolism pathways, including the TCA cycle and glycolysis/gluconeogenesis, across land use comparisons. These findings indicate that the environmental context could shape the organization and function of rhizosphere biochemical networks through coordinated shifts in bacterial community structure and metabolic pathway activity. The observed land-use-specific metabolic signatures suggest that land use changes trigger adaptive biochemical responses that may influence plant performance and ecosystem functioning. The integration of microbial community profiling with metabolomics provides a framework for understanding the mechanistic basis of plant–soil–microbe interactions across environmental gradients. Future research should incorporate temporal sampling designs covering all representative plants from the field and not experimental manipulations to establish causal relationships between microbial community dynamics and metabolite production in rhizosphere environments.

## Figures and Tables

**Table 1:** Rhizosphere soil microbial phenotypic structure dominance across land uses.

Microbial Ratios	Mean ± SE			F-Value	Significance	Pairwise Comparison (Tukey HSD)		
	F	E	G			F vs. E	E vs. G	G vs. F
Aerobe/Anaerobe	3.13 ± 0.08	3.11 ± 0.1	3.32 ± 0.11	1.16	ns	ns	ns	ns
G+/G−	0.4 ± 0.006	0.36 ± 0.009	0.29 ± 0.013	26.661	***	*	***	***
F/B	0.083 ± 0.001	0.085 ± 0.003	0.089 ± 0.003	1.455	ns	ns	ns	ns
F/PLFA	0.077 ± 0.002	0.077 ± 0.002	0.082 ± 0.003	1.417	ns	ns	ns	ns
B/PLFA	0.923 ± 0.002	0.085 ± 0.003	0.918 ± 0.003	1.417	ns	ns	ns	ns

ns—not significant (*p* > 0.05); *—*p* < 0.05; ***—*p* < 0.001.

**Table 2:** Co-inertia analysis parameters.

Land Use	Pairwise Co-Inertia	RV	First Axis Variance (%)	Strength
Grassland	Microbiota vs. Soil properties	0.51	48.3	*
	Metabolites vs. Soil properties	0.6	71.5	*
	Microbiota vs. Metabolites	0.5	62.8	*
Edge	Microbiota vs. Soil properties	0.81	79.2	**
	Metabolites vs. Soil properties	0.74	89.2	*
	Microbiota vs. Metabolites	0.72	84.9	*
Forest	Microbiota vs. Soil properties	0.68	58.2	*
	Metabolites vs. Soil properties	0.91	81.2	***
	Microbiota vs. Metabolites	0.71	66.2	*

*—*p* < 0.05; **—*p* < 0.01; ***—*p* < 0.001.

## Data Availability

Data are available throughout the manuscript.

## Supplementary Materials

The following supporting information can be downloaded at: https://www.mdpi.com/article/10.3390/metabo15110684/s1, Table S1. Soil physicochemical properties comparison across land uses; Table S2. Metabolomics pathway analysis of land uses specific differences in biochemical processes.

## References

[1] Bouwmeester H., Dong L., Wippel K., Hofland T., Smilde A. (2025). The chemical interaction between plants and the rhizosphere microbiome. Trends Pant Sci..

[2] Alawiye T.T., Babalola O.O. (2019). Bacterial diversity and community structure in typical plant rhizosphere. Diversity.

[3] Wankhade A., Wilkinson E., Britt D.W., Kaundal A. (2025). A Review of Plant–Microbe Interactions in the Rhizosphere and the Role of Root Exudates in Microbiome Engineering. Appl. Sci..

[4] Xue P., Minasny B., McBratney A.B. (2022). Land-use affects soil microbial co-occurrence networks and their putative functions. Appl. Soil Ecol..

[5] Wang D., Deng S., Yang H., Li N., Feng Q., Liu J., Yin H. (2025). The microbial network exhibits higher complexity in the rhizosphere than in bulk soils along elevational gradients in the alpine forests. Appl. Soil Ecol..

[6] Liu X., Fan X., Zhang M., Zhang H., Yue Y., Wu J., Teng W., Mu N., Teng K., Wen H. (2025). Insights into the interlinkages between rhizosphere soil extracellular enzymes and microbiome assemblages across soil profiles in grasslands. Appl. Soil Ecol..

[7] Kovacs E.D., Rusu T., Kovacs M.H. (2025). Sustainable soil volatilome: Discrimination of land uses through GC–MS-identified volatile organic compounds. Separations.

[8] Sokol N.W., Foley M.M., Blazewicz S.J., Bhattacharyya A., DiDonato N., Estera-Molina K., Firestone M., Greenlon A., Hungate B.A., Kimbrel J. (2024). The path from root input to mineral-associated soil carbon is dictated by habitat-specific microbial traits and soil moisture. Soil Biol. Biochem..

[9] Gupta V.V.S.R., Tiedje J.M. (2024). Ranking environmental and edaphic attributes driving soil microbial community structure and activity with special attention to spatial and temporal scales. mLife.

[10] Yusuf A., Li M., Zhang S.Y., Odedishemi-Ajibade F., Luo R.F., Wu Y.X., Zhang T.T., Yunusa Ugya A., Zhang Y., Duan S. (2025). Harnessing plant–microbe interactions: Strategies for enhancing resilience and nutrient acquisition for sustainable agriculture. Front. Plant Sci..

[11] Chauhan P., Sharma N., Tapwal A., Kumar A., Verma G.S., Meena M., Seth C.S., Swapnil P. (2025). Soil Microbiome: Diversity, Benefits and Interactions with Plants. Sustainability.

[12] Ma Y., Wang H., Kang Y., Wen T. (2025). Small molecule metabolites drive plant rhizosphere microbial community assembly patterns. Front. Microbiol..

[13] Chen Q., Song Y., An Y., Lu Y., Zhong G. (2024). Mechanisms and Impact of Rhizosphere Microbial Metabolites on Crop Health, Traits, Functional Components: A Comprehensive Review. Molecules.

[14] Fadiji A.E., Barmukh R., Varshney R.K., Singh B.K. (2023). Exploring the connectivity between rhizosphere microbiomes and the plant genes: A way forward for sustainable increase in primary productivity. J. Sustain. Agric. Environ..

[15] Luo C., He Y., Chen Y. (2025). Rhizosphere microbiome regulation: Unlocking the potential for plant growth. Curr. Res. Microb. Sci..

[16] Marfil-Santana M.D., Martínez-Cárdenas A., Ruíz-Hernández A., Vidal-Torres M., Márquez-Velázquez N.A., Figueroa M., Prieto-Davó A. (2021). A meta-omics analysis unveils the shift in microbial community structures and metabolomics profiles in mangrove sediments treated with a selective actinobacterial isolation procedure. Molecules.

[17] Wu G., Zhang M., Han P., Guo D., Shi Y., Mu D., Li X., Wu X. (2024). Microbial community succession patterns and metabolite profiles in cigar tobacco during different mildew stages. Ind. Crop. Prod..

[18] Reinula I., Träger S., Järvine H.T., Kuningas V.M., Kaldra M., Aavik T. (2024). Beware of the impact of land use legacy on genetic connectivity: A case study of the long-lived perennial *Primula veris*. Biol. Conserv..

[19] Luo X., Tong Z., Xie Y., An R., Yang Z., Liu Y. (2022). Land Use Change under Population Migration and Its Implications for Human–Land Relationship. Land.

[20] Nirhamo A., Aakala T., Kouki J. (2025). Forest biodiversity in boreal Europe: Species richness and turnover among old-growth forests, managed forests and clearcut sites. Biol. Conserv..

[21] Kovacs E.D., Kovacs M.H., Barcelo D., Pereira P. (2024). Nonsteroidal anti-inflammatory drugs impact the microbial community in three different soil types—A laboratory experiment. Case Stud. Chem. Environ. Eng..

[22] Kovacs E.D., Silaghi-Dumitrescu L., Roman C., Tian D. (2022). Structural and metabolic profiling of Lycopersicon esculentum rhizosphere microbiota artificially exposed at commonly used non-steroidal anti-inflammatory drugs. Microorganisms.

[23] Blight E.G., Dyer W.J. (1959). A rapid method of total lipid extraction and purification. Can. J. Biochem. Physiol..

[24] Lai Z., Tsugawa H., Wohlgemuth G., Mehta S., Mueller M., Zheng Y., Ogiwara A., Meissen J., Showalter M., Takeuchi K. (2018). Identifying metabolites by integrating metabolome databases with mass spectrometry cheminformatics. Nat. Methods.

[25] Baquer G., Semente L., Rafols P., Martin-Saiz L., Bookmeyer C., Fernandez J.A., Correig X., Garcia-Altares M. (2023). rMSIfragment: Improving MALDI-MSI lipidomics through automated in-source fragment annotation. J. Cheminform..

[26] Peris-Diaz M.D., Sweeney S.R., Rodak O., Sentandreu E., Tiziani S. (2019). R-MetaboList 2: A flexible tool for metabolite annotation from high-resolution data-independent acquisition mass spectrometry analysis. Metabolites.

[27] Gul S., Whalen J.K. (2022). Chapter six—Perspectives and strategies to increase the microbial-derived soil organic matter that persists in agroecosystems. Adv. Agron..

[28] Samaniego T., Ramirez J., Solórzano R. (2024). Litter Decomposition Rates of Four Species of Agroecological Importance in the Peruvian Coast and Andean Highland. Nitrogen.

[29] Wegner R., Plassmann M., Sauerland L., Carter A., Monteux S., Oburger E., Wild B. (2025). Back to the roots: Characterizing root exudates of dominant tundra plants to improve the understanding of plant-soil interactions in a changing arctic. Soil Biol. Biochem..

[30] Shen X., Yang F., Xiao C., Zhou Y. (2020). Increased contribution of root exudates to soil carbon input during grassland degradation. Soil Biol. Biochem..

[31] Maciel-Rodríguez M., Moreno-Valencia F.D., Plascencia-Espinosa M. (2025). The Role of Plant Growth-Promoting Bacteria in Soil Restoration: A Strategy to Promote Agricultural Sustainability. Microorganisms.

[32] Du X.F., Liu H.W., Li Y.B., Li B., Han X., Li Y.H., Mahamood M., Li Q. (2022). Soil community richness and composition jointly influence the multifunctionality of soil along the forest-steppe ecotone. Ecol. Indic..

[33] Marfo T.D., Datta R., Vranova V., Ekielski A. (2019). Ecotone dynamics and stability from soil perspective: Forest-Agriculture land transition. Agriculture.

[34] Ortiz-Colin P., Hulshof C.M. (2024). Ecotones as Windows into Organismal-to-Biome Scale Responses across Neotropical Forests. Plants.

[35] Aslan C.E., Zachmann L., Epanchin-Niell R.S., Brunson M.W., Veloz S., Sikes B.A. (2022). Soil characteristics and bare ground cover differ among jurisdictions and disturbance histories in Western US protected area-centered ecosystems. Front. Ecol. Evol..

[36] Canarini A., Kaiser C., Merchant A., Richter A., Wanek W. (2019). Root exudation of primary metabolites: Mechanisms and their roles in plant responses to environmental stimuli. Front. Plant Sci..

[37] Dussarrat T., Latorre C., Barros Santos M.C., Aguado-Norese C., Prigent S., Díaz F.P., Rolin D., González M., Müller C., Gutiérrez R.A. (2025). Rhizochemistry and soil bacterial community are tailored to natural stress gradients. Soil Biol. Biochem..

[38] Li S., Ahmed W., Jiang T., Yang D., Yang L., Hu X., Zhao M., Peng X., Yang Y., Zhang W. (2025). Amino acid metabolism pathways as key regulators of nitrogen distribution in tobacco: Insights from transcriptome and WGCNA analyses. BMC Plant Biol..

[39] Zhang J., Chen G., Zhao P., Zhou Q., Zhao X. (2017). The abundance of certain metabolites responds to drought stress in the highly drought tolerant plant *Caragana korshinskii*. Acta Physiol. Plant..

[40] Walker R.P., Chen Z.H., Famiani F. (2021). Gluconeogenesis in Plants: A Key Interface between Organic Acid/Amino Acid/Lipid and Sugar Metabolism. Molecules.

[41] Nunes-Nesi A., Fernie A.R., Stitt M. (2010). Metabolic and signaling aspects underpinning the regulation of plant carbon nitrogen interactions. Mol. Plant.

[42] Shtaida N., Khozin-Goldberg I., Boussiba S. (2015). The role of pyruvate hub enzymes in supplying carbon precursors for fatty acid synthesis in photosynthetic microalgae. Photosynth. Res..

[43] Jardine K.J., Honeker L.K., Zhang Z., Kwatcho Kengdo S., Yang Y., Roscioli J., Riley W.J. (2025). Evolutionary and functional relationships between plant and microbial C1 metabolism in terrestrial ecosystems. New Phytol..

[44] Li Z., Xu X., Yang K., Zhu C., Liu Y., Gao Z. (2022). Multifaceted analyses reveal carbohydrate metabolism mainly affecting the quality of postharvest bamboo shoots. Front. Plant Sci..

[45] Chen L., Meng Y., Bai Y., Yu H., Qian Y., Zhang D., Zhou Y. (2023). Starch and sucrose metabolism and plant hormone signaling pathways play crucial roles in Aquilegia salt stress adaptation. Int. J. Mol. Sci..

[46] Stincone A., Prigione A., Cramer T., Wamelink M.M.C., Campbell K., Cheung E., Olin-Sandoval V., Grüning N.M., Krüger A., Tauqeer Alam M. (2015). The return of metabolism: Biochemistry and physiology of the pentose phosphate pathway. Biol. Rev..

[47] Kruger N.J., Von Schaewen A. (2003). The oxidative pentose phosphate pathway: Structure and organization. Curr. Opin. Plant Biol..

[48] Ma Q., Xu M., Liu M., Cao X., Hill P.W., Chadwick D.R., Wu L., Jones D.L. (2022). Organic and inorganic sulfur and nitrogen uptake by co-existing grassland plant species competing with soil microorganisms. Soil Biol. Biochem..

[49] Nikiforova V.J., Kopka J., Tolstikov V., Fiehn O., Hopkins L., Hawkesford M.J., Hesse H., Hoefgen R. (2005). Systems Rebalancing of Metabolism in Response to Sulfur Deprivation, as Revealed by Metabolome Analysis of Arabidopsis Plants. Plant Physiol..

[50] Mondal S., Karmakar S., Panda D., Pramanik K., Bose B., Singhal R.K. (2023). Crucial plant processes under heat stress and tolerance through heat shock proteins. Plant Stress.

[51] Lv J., Yang S., Zhou W., Liu Z., Tan J., Wei M. (2024). Microbial regulation of plant secondary metabolites: Impact, mechanisms and prospects. Microbiol. Res..

[52] Marks B.B., Nogueira M.A., Hungria M. (2025). Microbial Secondary Metabolites and Their Use in Achieving Sustainable Agriculture: Present Achievements and Future Challenges. Agronomy.

[53] Zhang T., Dong X., Yang J., Li Z., Zhu J. (2025). Effects of near-natural forest management on soil microbial communities in the temperate-subtropical transition zone in China. Microorganisms.

[54] Vivian J., Chazdon R.L., Catling A.A., Lee D.J. (2025). Early evidences of links between soil microbes and forest restoration through multiple ecosystem metrics. Front. For. Glob. Change.

[55] Buschmann H., Edwards P.J., Dietz H. (2005). Variations in growth pattern and response to slug damage among native and invasive provenances of four perennial Brassicaceae species. J. Ecol..

[56] Chen H., Zhao X., Lin Q., Li G., Kong W. (2019). Using a combination of PLFA and DNA-based sequencing analyses to detect shifts in the soil microbial community composition after a simulated spring precipitation in a semi-arid grassland in China. Sci. Total Environ..

[57] Li J., Li Y.T., Yang X.D., Zhang J.J., Lin Z.A., Zhao B.Q. (2015). Microbial community structure and functional metabolic diversity are associated with organic carbon availability in an agricultural soil. J. Integr. Agric..

[58] El-Azaz J., Moore B., Maeda H.A. (2025). Re-tuning of aromatic amino acid biosynthesis before and after the evolution of tyrosine-derived grass lignin. Plant Physiol..

[59] Cannell N., Emms D.M., Hetherinton A.J., MacKay J., Kelly S., Dolan L., Sweetlove L.J. (2020). Multiple metabolic innovations and losses are associated with major transitions in land plant evolution. Curr. Biol..

