# Supplementary material for: Land Use Shapes the Rhizosphere Microbiome and Metabolome of Naturally Growing Barbarea vulgaris
Source: Metabolites. 2025 Oct 22;15(11):684. doi: 10.3390/metabo15110684 (PMC12654609; doi:10.3390/metabo15110684)
Supplement: Supplementary file 1 [file metabolites-15-00684-s001.zip › metabolites-3884426-supplementary.pdf]

metabolites

Article

Land use shapes naturally grown Barbarea vulgaris rhizosphere microbiome and metabolome

Firstname Lastname [1], Firstname Lastname [2] and Firstname Lastname [2,*]

Supplementary material. Table S1. Soil physicochemical properties comparison across land uses

Soil properties	G	E	F	$F_{2,42}$	p-val	η2	Tukey HSD			Pattern
							G vs E	E vs F	G vs F	
pH	6.5 ± 0.05	6.3 ± 0.03	5.9 ± 0.04	61.23	0.000	0.745	***	***	***	G > E > F
SOM %	7.5 ± 0.19	6.9 ± 0.13	5.2 ± 0.15	57.15	0.000	0.731	*	***	***	G > E > F
Calcium (mg·kg-1)	588.5 ± 3.4	586.1 ± 6.8	577.1 ± 4.6	1.39	0.26	0.062	ns	ns	ns	G ≈ E ≈ F
Magnesium (mg·kg-1)	298 ± 4.9	303.4 ± 7.6	283.2 ± 3.9	3.35	0.045	0.138	ns	ns	*	G ≈ E ≈ F
Potassium (mg·kg-1)	210.8 ± 6.1	193.1 ± 5.5	178.1 ± 3.0	10.54	0.000	0.334	*	***	ns	G > E ≈ F
Sodium (mg·kg-1)	31.2 ± 1.5	25.9 ± 1.1	27.9 ± 1.7	3.27	0.048	0.135	*	ns	ns	G > E ≈ F
Chlorine (mg·kg-1)	24.7 ± 1.3	21.5 ± 1	24.6 ± 1.1	2.56	0.089	0.109	ns	ns	ns	G ≈ E ≈ F
Ammonium (mg·kg-1)	21.8 ± 1.3	22.4 ± 0.7	18.7 ± 0.7	4.52	0.017	0.177	ns	ns	*	G ≈ E ≈ F
Nitrate (mg·kg-1)	8.63 ± 0.26	6.15 ± 0.31	2.63 ± 0.22	125.7	0.000	0.857	***	***	*	G > E > F

 https://doi.org/10.3390/xxxxx

Supplementary material. Table S2. Metabolomics pathway analysis of land uses specific differences in biochemical processes

**Comparison**	**No.**	**Corresponding pathway name**	***p*-val**	**FDR**	**Impact**
Grassland *vs*. Edge	1	Alanine, aspartate and glutamate metabolism	0.006	0.146	0.3325
	2	beta-Alanine metabolism	0.033	0.356	0.3993
	3	Pyrimidine metabolism	0.002	0.106	0.0375
	4	Lysine degradation	0.007	0.146	0.1227
	5	Arginine biosynthesis	0.015	0.2	0.1223
	6	Glycolysis/Gluconeogenesis	0.049	0.431	0.1459
	7	Phenylalanine metabolism	0.106	0.643	0.2381
	8	Glycerolipid metabolism	0.2	0.891	0.2368
	9	Nitrogen metabolism	0.003	0.106	0
	10	Cysteine and methionine metabolism	0.010	0.153	0.0209
	11	Pantothenate and CoA biosynthesis	0.244	0.930	0.0476
Edge *vs*. Forest	1	Citrate cycle (TCA cycle)	0.000	0.001	0.3171
	2	Alanine, aspartate and glutamate metabolism	0.000	0.000	0.2019
	3	Glycine, serine and threonine metabolism	0.078	0.330	0.4744
	4	beta-Alanine	0.135	0.496	0.3993
	5	Glyoxylate and dicarboxylate metabolism	0.002	0.048	0.2200
	6	Arginine biosynthesis	0.008	0.073	0.2394
	7	Glycolysis/Gluconeogenesis	0.007	0.073	0.2843
	8	Pyruvate metabolism	0.005	0.073	0.1927
	9	Lysine degradation	0.612	1	0.1227
	10	Glycerolipid metabolism	0.085	0.339	0.2508
	11	Phenylalanine metabolism	0.222	0.657	0.2381
	12	Pyrimidine metabolism	0.006	0.073	0.1169
	13	Ascorbate and aldarate metabolism	0.002	0.048	0
	14	Galactose metabolism	0.008	0.073	0.0412
	15	Cysteine and methionine metabolism	0.017	0.103	0.0693
	16	Amino sugar and nucleotide sugar metabolism	0.038	0.2	0.1071
	17	Pentose phosphate pathway	0.156	0.520	0.1588
Grassland *vs*. Forest	1	Citrate cycle (TCA cycle)	0.000	0.005	0.2873
	2	Alanine, aspartate and glutamate metabolism	0.000	0.000	0.1995
	3	Glycine, serine and threonine metabolism	0.054	0.253	0.4744
	4	beta-Alanine	0.104	0.416	0.3993
	5	Glyoxylate and dicarboxylate metabolism	0.001	0.032	0.2200
	6	Glycolysis/Gluconeogenesis	0.004	0.082	0.2843
	7	Pyruvate metabolism	0.021	0.151	0.1927
	8	Arginine biosynthesis	0.051	0.253	0.2394
	9	Glycerolipid metabolism	0.065	0.272	0.2508
	10	Phenylalanine metabolism	0.193	0.617	0.2381
	11	Pentose phosphate pathway	0.121	0.436	0.1588
	12	Amino sugar and nucleotide sugar metabolism	0.023	0.151	0.1071
	13	One carbon pool by folate	0.029	0.179	0.1074
	14	Cysteine and methionine metabolism	0.010	0.121	0.0693
	15	Starch and sucrose metabolism	0.011	0.121	0.0600
	16	Lysine degradation	0.555	1	0.1227
	17	Butanoate metabolism	0.006	0.100	0.0318
